# Development of a DNA-based real-time PCR assay for the quantification of *Colletotrichum camelliae* growth in tea (*Camellia sinensis*)

**DOI:** 10.1186/s13007-020-00564-x

**Published:** 2020-02-17

**Authors:** Shengnan He, Huchen Chen, Yi Wei, Tai An, Shouan Liu

**Affiliations:** Laboratory of Molecular Plant Pathology, College of Plant Science, Jilin University, Changchun, Jilin People’s Republic of China

**Keywords:** Tea plant (*camellia sinensis*), *Colletotrichum camelliae*, *Colletotrichum* spp., qRT-PCR

## Abstract

**Background:**

Tea, which is produced from new shoots of existing tea plants (*Camellia sinensis*), is one of the most popular, non-alcoholic, healthy beverages worldwide. *Colletotrichum camelliae* is one of the dominant fungal pathogens of tea. The interaction of *C. camelliae* with tea could be a useful pathosystem to elucidate various aspects of woody, medicinal plant-fungal interactions. Currently, many studies characterizing resistance or virulence and aggressiveness use lesion size at the infection sites on the leaves to quantify the growth of the pathogen. However, this method does not offer the sensitivity needed for the robust quantification of small changes in aggressiveness or the accurate quantification of pathogen growth at the early stages of infection.

**Results:**

A quantitative real-time polymerase chain reaction (qRT-PCR) assay was developed for the quantification of *C. camelliae* growth on tea plant. This method was based on the comparison of fungal DNA in relation to plant biomass. This assay was used to investigate the phenotypes of tea plant cultivars in response to *C. camelliae* infection. Two cultivars, Zhongcha 108 (ZC108) and Longjing 43 (LJ43), were tested with this method. ZC108 was previously reported as an anthracnose-resistant cultivar against *C. camelliae*, while LJ43 was susceptible. The traditional lesion measurement method showed that both cultivars were susceptible to a virulent strain of *C. camelliae*, while the qRT-PCR approach indicated that very little fungal growth occurred in the anthracnose-resistant cultivar ZC108. The observed results in this study were consistent with previously published research. In addition, the DNA-based real-time PCR method was applied for analysis of pathogenic differences in general *C. camelliae* isolates and among several *Colletotrichum* spp that infect tea.

**Conclusions:**

This study showed that the DNA-based qRT-PCR technique is rapid, highly sensitive and easily applicable for routine experiments and could be used in screening for resistant tea plant cultivars or to identify differences in pathogen aggressiveness within and among *Colletotrichum* species.

## Introduction

Tea (*Camellia sinensis*) is one of the most economically important crops in the world. Tea is widely grown in Asian, African and South American countries to produce non-alcoholic healthy beverages worldwide. Tea has also been used medicinally in China over a long period of time [1]. Investigations into the medicinal uses of tea have been intense, and now, many medicinal products have been developed [1–3]. Tea is a perennial, evergreen woody plant, and some plants have lived for more than a thousand years. During its long life, tea faces many biotic and abiotic stresses, including pathogens, insects, low and high temperatures, and heavy metals [4–12]. Therefore, tea can be used as a perennial, medicinal species to characterize how defence responses are activated or suppressed.

Pathogens of tea include *Colletotrichum* spp., a large genus of Ascomycete fungi causing anthracnose disease on a wide range of host genera [13–19]. *Colletotrichum camelliae*, *C. fructicola*, *C. siamense* and *C. fioriniae* have been isolated in the main tea growing region of China and caused anthracnose on *Ca. sinensis* [20–23]. *Colletotrichum camelliae* and *C. fructicola* were the species most often isolated and were proposed as dominant pathogens of tea [20–23]. *Colletotrichum camelliae* damages tea leaves and causes several tea diseases known as tea leaf blight, tea brown blight, or tea anthracnose [10, 21–24]. The fungus *C. fructicola* is both a pathogen and an endophyte of several plant species, while *C. camelliae* was only isolated as a pathogen of *Ca. sinensis* in previous studies [22, 23]. The interaction of tea and *C. camelliae* would be a useful pathosystem to elucidate various aspects of woody medicinal plant-fungi interactions. In addition, *C. siamense* was originally isolated from coffee (*Coffea arabica*) berries of Thailand and also caused tea anthracnose in China [22], while *C. fioriniae* had been isolated from various hosts, including *Camellia* spp. grown in Yunnan, Fujian, Sichuan, and Zhejiang Province of China [22].

The easiest way to evaluate plant resistance or susceptibility is to score the severity of visual disease symptoms during the plant-fungus interaction. Visible lesions often occurs late in this host–pathogen interaction, making it difficult to correctly measure lesion size at early growth stages. In addition, when lesion sizes show minor differences during tea cultivar interactions with diverse pathogen isolates, the lesion measurement method may not be adequate. An alternative approach for quantifying fungal growth is available through the use of a highly sensitive DNA-based methods such as quantitative real-time PCR (qRT-PCR). Those methods include two types of procedures. In one procedure, qRT-PCR was based on only the DNA of the fungi to measure the growth of the pathogens [25, 26]. However, that method does not consider the normalization of pathogenic DNA in relation to plant DNA biomass. Therefore, a second method that considers both plant and pathogen interaction has been developed for the precise measurement of pathogen growth in various host plants [27–31]. In the case of the tea plant-*C. camelliae* interaction, the visual lesion measurement assay has remained in use thus far despite certain disadvantages as indicated above.

Currently, genes for actin (ACT) and β-tubulin (TUB) as well as the internal transcribed spacer (ITS) region of ribosomal RNA, and ribosomal rDNA are often used as standards for the quantification of fungal biomass [25, 27, 30]. In addition, the genes encoding fungal cutinase (CutA), plasma membrane ATPase (PMA) and GDSL-like lipase (GLL) have been used for qRT-PCR in certain plant-fungi interaction systems [25, 29, 31]. Some of those are involved in basic cellular processes, primary metabolism or cell structure maintenance [32]. For *C. higginsianum* and *C. gloeosporioides*, the ACT and ITS, respectively, have been widely used to quantify and detect fungal growth in host plants [27, 33]. To date, no such information has been reported on tea plant pathogens like *C. camelliae*, *C. fructicola*, *C. siamense* and *C. fioriniae*.

This study reports on the optimization of a qRT-PCR-based analysis for *C. camelliae* quantification during its interaction with tea plant. This DNA-based methodology could be applied in two ways in the future: (i) to compare tea germplasm responses to *C. camelliae* and to detect resistant or susceptible tea plant cultivars and (ii) to quantify pathogenic differences in general *C. camelliae* assays and among isolates of several *Colletotrichum* spp.

## Results

### DNA-based analysis of real-time PCR primers for quantification of *C. camelliae* and tea

To quantify *C. camelliae* or tea DNA, qRT-PCR primers were designed to efficiently amplify the target sequences, respectively (Table 1). The targets were Cs18SrDNA1 in tea and glyceraldehyde-3-phosphate dehydrogenase (GAPDH) in *C. camelliae*. Both targets are conserved and have been used as reference genes for plants or fungi [21–23, 34]. The ITS region of ribosomal RNA from *Colletotrichum* spp. was also included as it has often been used in development of PCR primers for the detection of various fungi [26]. The primer pair S37/S38 was designed based on the Cs18SrDNA1 sequence and was expected to amplify a 167-bp DNA fragment from tea [34]. The primer pairs S572/S573 and S576/S577 were designed based on the GAPDH and ITS sequences of *C. camelliae*, respectively (Table 1) [21, 22]. These primer pairs were expected to amplify an 82-bp and a 62-bp DNA fragment of *C. camelliae*, respectively.

**Table 1:** Primers designed for quantitative real-time PCR

Primer names	Reference gene	GenBank Accession Number	Primer sequence (5′-3′) forward/reverse	Product Tm (°C)	Amplicon size (bp)	GC (%)	qRT-PCR efficiency (%)
S37/S38	Cs18S rDNA1	AY563528.1	GACTCCGCTGGCACCTTAT/GCCCTTCCGTCAATTCCT	83.7	167	48	97.6–98.1
S572/S573	GAPDH	KJ954782	CCCGCATCTGGTAGACAAGA/TGATAGCATGTGTCCCTCCG	81.0	82	50	92.0–98.8
S576/S577	ITS	KJ955081	AAAGGTAGTGGCGGACCCT/CCCAGTGCGAGACGTAAAGT	82.5	62	58	96.0–97.6

*Tm* melting temperature, *GC (%)* guanine-cytosine percentages

To guarantee the amplification of a specific DNA region of *C. camelliae* for the quantification of fungal biomass with those primers, DNAs from *Colletotrichum* spp. and several other tea pathogens isolated from different tea gardens in China were first tested (Additional file 1: Table S1). Those fungal pathogens include, *C. camelliae* isolates (CCA, CCB, LS_19, ZJ1A5, ZJ1A8, HB1A4), *C. fructicola* (SX_6), *C. siamense* (E-8-1), *C. fioriniae* (ZJ1A2), *Pseudopestalotiopsis camelliae-sinensis* (HUN1A3) and a *Neopestalotiopsis* sp. (YN1A5) [21–23, 35]. The fungal pathogens, *P. camelliae-sinensis* and *Neopestalotiopsis* sp., cause gray blight disease in tea, resulting in severe tea yield losses [35]. Recent geographical distribution and pathogenicity tests indicated that *P. camelliae-sinensis* was the dominant cause of gray blight of tea in China [35]. In addition, the rice blast fungus *Magnaporthe oryzae* was also included as a non-tea plant pathogen control [36].

The initial experiment was run with 27 ng of DNA for each technical replicate. The specificity of the primers for all the proposed genes was tested by conventional PCR on 2.0% agarose gels (Additional file 2: Figure S1). Specific amplification of the GAPDH PCR product could be observed only in the samples of *C. camelliae* isolates, which included isolates CCA, CCB, LS_19, ZJ1A5, ZJ1A8 and HB1A4 (Additional file 2: Figure S1a). For the other fungal samples, DNA products were not observed (Additional file 2: Figure S1a). This indicated that the GAPDH primer specifically quantified *C. camelliae.* The amplification of the ITS PCR product was observed for *C. camelliae, C. fructicola*, *C. siamense* and *C. fioriniae* (Additional file 2: Figure S1b). However, the ITS primers designed in this study did not amplify the DNA from *P. camelliae-sinensis*, *Neopestalotiopsis *sp. or *M. oryzae*. These results indicated that the ITS primer could quantify the *Colletotrichum* spp. including *C. camelliae*,* C. fructicola*, *C. siamense* and *C. fioriniae*. Next, the *C. camelliae* isolate CCA was selected and further tested for its growth on tea plants as measured by qPCR using the GAPDH and ITS primers.

**Fig. 1:**
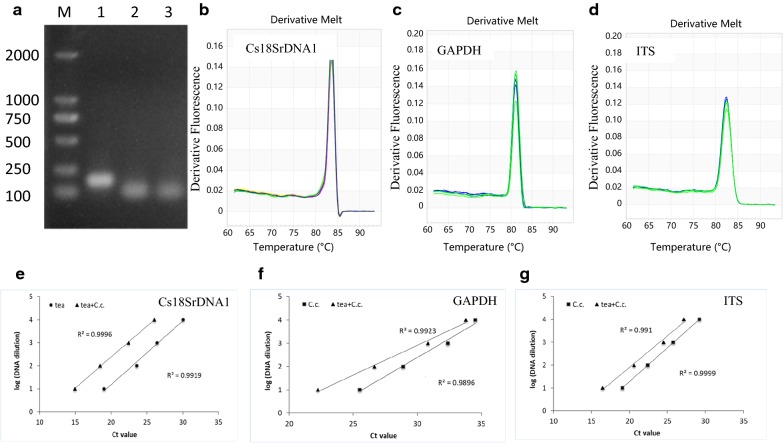
Validation of primers for biomass quantification of tea plant and pathogen. **a** Amplification results for three genes using *C. camelliae* (*C.c*)-infected tea plant DNA*.* DNA. M: DL2000 Marker, 1: Cs18SrDNA1, 2: GAPDH, 3: ITS. **b**–**d** Melting curve analyses of three genes. Each qRT-PCR product had a single melt curve indicating the breakdown of only one PCR product. **b** Cs18SrDNA1, **c** GAPDH, **d** ITS. **e**–**g** The primer efficiency for the PCR quantification of the gene was determined using a serial dilution of DNA templates from tea plant, *C. camelliae* (*C.c*), and *C. camelliae*-infected tea plant (tea + *C.c*). **e** Cs18SrDNA1, **f** GAPDH, **g** ITS. The respective correlation coefficients (R^2^) are indicated

To assess the specificity of the amplification of the target DNA regions of the pathogen and plant biomass, DNAs were further extracted from the 2-year-old healthy tea plants of the cultivar Longjing 43 (LJ43), *C. camelliae* CCA, and from *C. camelliae* CCA-infected leaves of LJ43. For all the genes, a single band of the expected size was obtained, indicating that no primer dimers or nonspecific amplified products had been generated (Fig. 1a). To confirm the specificity of the primers, the melting curves for all tested sequences were established by qRT-PCR. The melt curves observed for all the sequences showed single well-defined sharp peaks. The single peak in the melt curves demonstrated the specificity of the annealing temperature and showed that the evaluated primers successfully amplified the desired amplicons (Fig. 1b–d). The melting temperature (Tm) values for the qRT-PCR products from the different genes ranged from 81.0 to 83.7 °C (Table 1). Those products showed good agreement with the published lengths and the guanine-cytosine (GC) percentages of the amplicons [22, 32].

In addition, the primer efficiency was tested using a tenfold dilution series of pure *C. camelliae* DNA, tea plant DNA and *C. camelliae*-infected tea plant DNA. For all the DNA samples, the primers yielded a linear amplification over the range of template concentrations with a correlation coefficient of R^2^ > 0.99 (Fig. 1e–g). At the same DNA concentration, the cycle quantification (Cq) value of ITS was approximately 5.5–7.5 cycles greater than that of GAPDH, indicating that the amplification of the ITS product was approximately 50–200 times more efficient than that of GAPDH. This suggested that the ITS primer was more sensitive than the primers developed based on the GAPDH gene and could be used to detect very low DNA concentrations. Overall, all the primer pairs were suitable for the quantification of the target genes even when using low input quantities of DNA.

### DNA-based real-time PCR for the quantification of *C. camelliae* growth after infection on tea

**Fig. 2:**
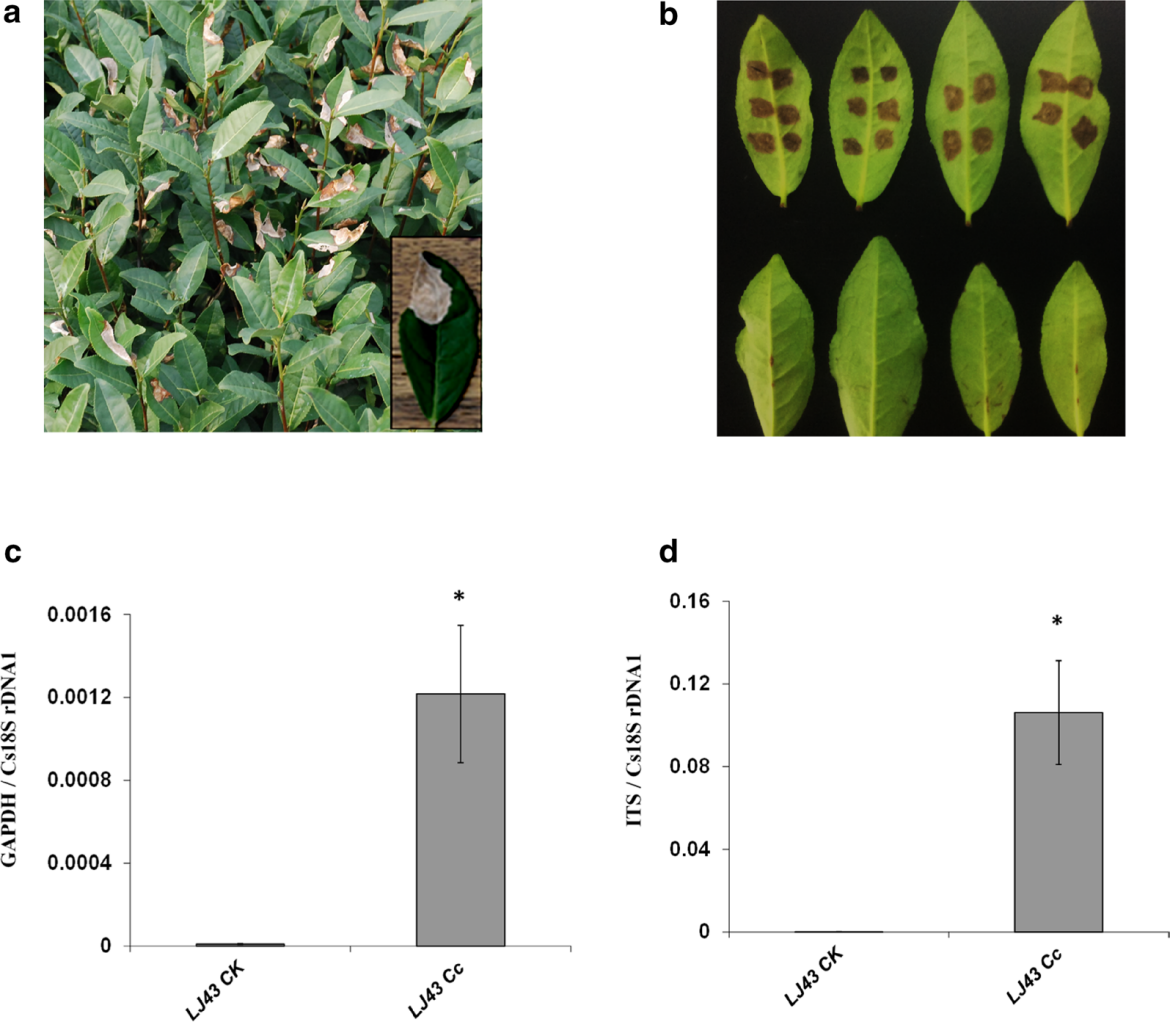
qRT-PCR methodology to quantify *C. camelliae* growth rate following infection of tea plant leaves. **a** Photo of tea plant cultivar LJ43 infested with *C. camelliae* in a tea garden. The close-up frame shows a single leaf with typical anthracnose symptoms. **b** Phenotypes of LJ43 leaves in response to *C. camelliae* CCA (Cc, top row) and ddH_2_O control (CK, bottom row). The leaves were wounded with a razor blade and immediately inoculated with 5 µL *C. camelliae* CCA spores (1 * 10^6^ spores mL^−1^). For the control, ddH_2_O alone was used. **c**, **d** qPCR-based biomass of *C. camelliae* CCA growth on tea plant LJ43. **c** The ratio of the primer pairs GAPDH/Cs18SrDNA1 was used to determine the fungal biomass. **d** The ratio of the primer pairs ITS/Cs18SrDNA1 was used to determine the fungal biomass. *P < 0.05 by the LSD test

The tea cultivar Longjing 43 (LJ43) was observed to be susceptible to *C. camelliae* in the tea garden (Fig. 2a) [10]. In this study, *C. camelliae* CCA growth was first quantified on the tea cultivar LJ43. Droplet inoculation of mechanically wounded tea leaves with *C. camelliae* isolate CCA spores was performed, and leaf samples were collected at 4 DPI (days post infection). To detect possible contamination by endophytic fungus from the tea, mechanically wounded leaves were incubated with water (ddH_2_O) as a control. As shown in Fig. 2b, lesions developed on leaves inoculated with CCA spores and their diameters were large at 4 DPI, while the un-inoculated control leaves did not show any lesion development, confirming that LJ43 was susceptible to *C. camelliae* CCA, as previously reported [10]. The same results were observed by analysing the pathogen growth with qRT-PCR (Fig. 2c, d). The amplification ratio of GAPDH/Cs18SrDNA1 or ITS/Cs18SrDNA1 was significantly higher in *C. camelliae*-infected tea leaves than in un-inoculated control tea leaves. These results indicated that the DNA-based qPCR method could be used to quantify *C*. *camelliae* CCA growth in tea.

**Fig. 3:**
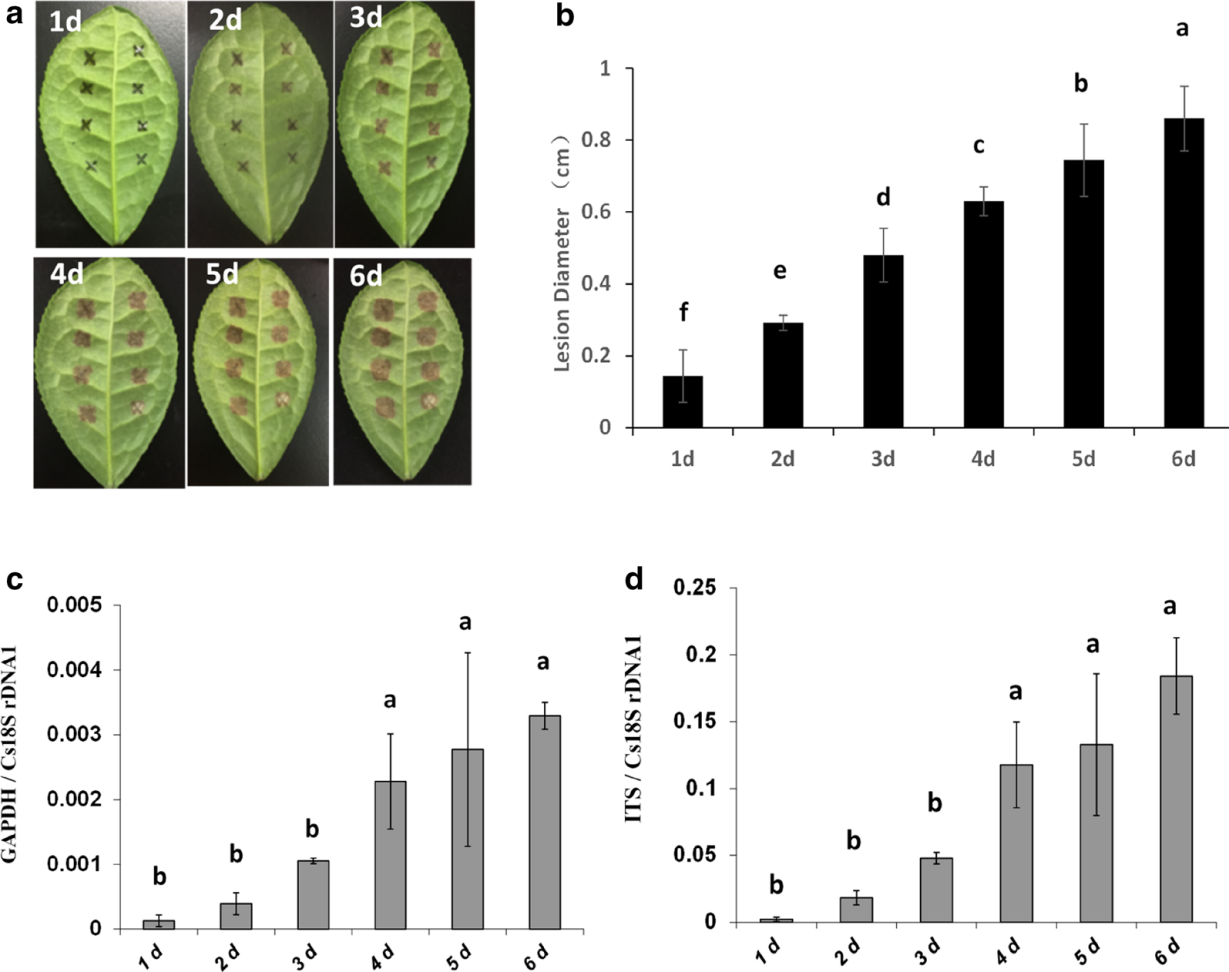
Comparative analysis of the two quantification methods for *C. camelliae* CCA growth over the time course of infection. **a** Photos of anthracnose leaf symptoms developed from 1 to 6 days after inoculation on tea cultivar LJ43. **b** The growth of *C. camelliae* CCA on tea plant was determined by the classical visual lesion measurement method over a time course of 6 days. **c**, **d** qRT-PCR-based biomass of *C. camelliae* CCA growth over 6 days. **c** The ratio of the primer pairs GAPDH/Cs18SrDNA1 was used to determine the fungal biomass. **d** The ratio of the primer pairs ITS/Cs18SrDNA1 was used to determine the fungal biomass. The letters represent significant differences at different times by LSD test (*P* < 0.05)

The quantification of fungal growth over time is often used to demonstrate differences in defence mechanisms at certain stages of the host-parasite interaction. To show that qRT-PCR-based quantification of *C. camelliae* is also suitable for temporal studies, the samples of *C. camelliae* CCA-infected LJ43 plants were collected at different times. Disease symptoms were observed and measured over time (Fig. 3a). The lesions grew, and the lesion diameter increased from 0.1 cm at 1 day to 0.8 cm at 6 DPI (Fig. 3b). This indicated that lesion size increased with time. The DNA-based qPCR results indicated that the fungal DNA increased in 3 days and reached a high level at 3 days compared to 1 day (Fig. 3c, d). It then dramatically increased from 3 to 4 days (more than twofold) and then slightly increased from 4 to 6 days (Fig. 3c, d). The qPCR data showed two stages of fungal growth and a clear disease development curve during the host–pathogen interaction (Fig. 3c, d), which was not observed with the lesion measurement method (Fig. 3b). These results indicated that the DNA-based qPCR assay can be used to examine increases in fungal biomass over time.

### DNA-based real-time PCR applied for analysis of *C. camelliae* growth on different tea cultivars

**Fig. 4:**
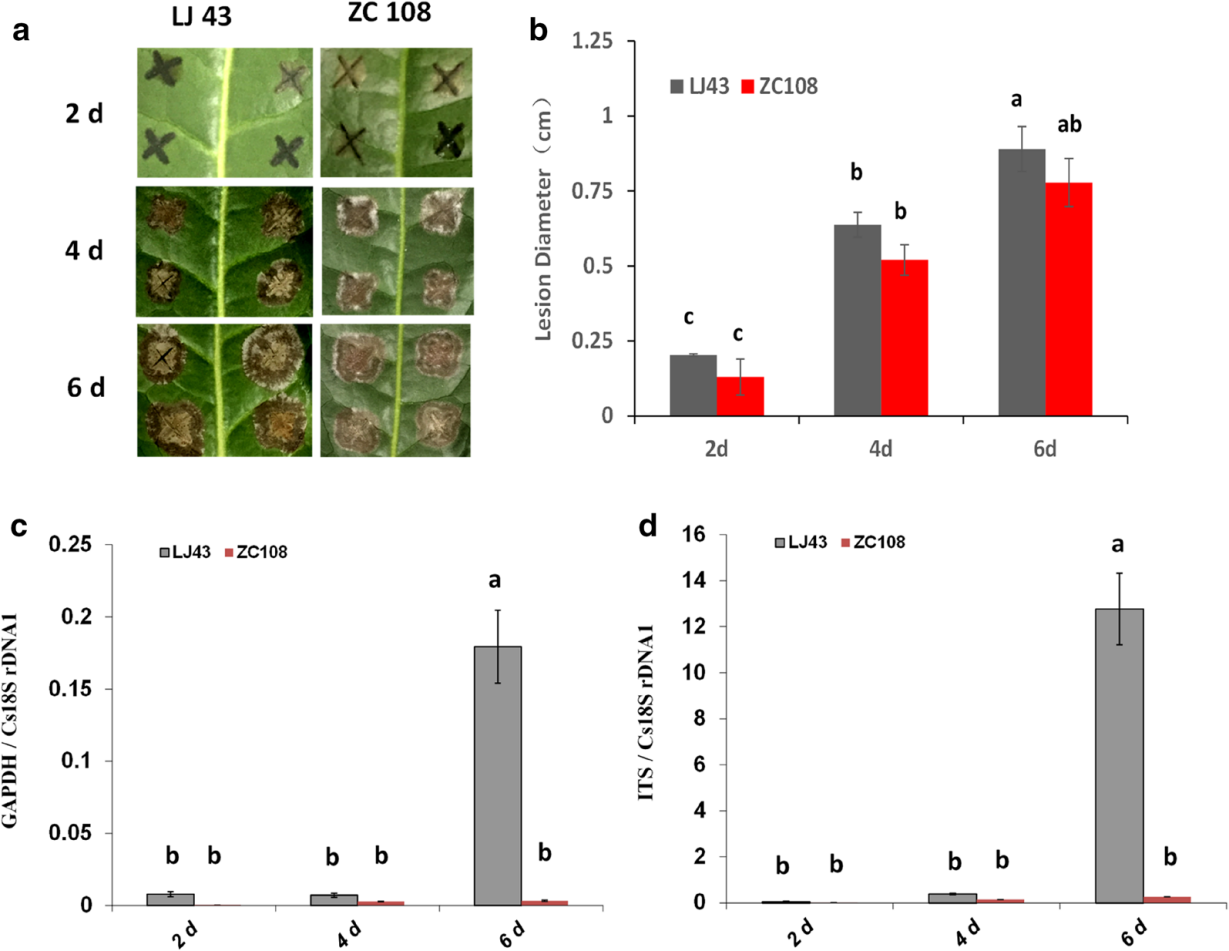
Quantification of *C. camelliae* CCA on tea plant cultivars LJ43 and ZC108. **a** Photos of anthracnose leaf symptoms developed on LJ43 and ZC108 over a time course of 2 days, 4 days and 6 days post *C. camelliae* CCA inoculation. **b** The growth of *C. camelliae* CCA on LJ43 and ZC108 was determined by visual lesion measurement. **c**, **d** qRT-PCR based biomass of the *C. camelliae* CCA growth on tea plants LJ43 and ZC108, respectively. **c** The ratio of the primer pairs GAPDH/Cs18SrDNA1 was used to determine the fungal biomass. **d** The ratio of the primer pairs ITS/Cs18SrDNA1 was used to determine the fungal biomass. The letters a and b represent significant differences between different samples according to LSD test (*P* < 0.05)

The responses of different tea cultivars to *C. camelliae* CCA were next evaluated. Two tea plant cultivars, LJ43 and Zhongcha 108 (ZC108), were used. Previous research reported that ZC108 was resistant to *C. camelliae* [10]. In this study, leaves of both LJ43 and ZC108 were droplet-inoculated with the *C. camelliae* isolate CCA. As shown in Fig. 4a, b, the lesion sizes increased with time on both CCA-inoculated LJ43 and ZC108 tea leaves. Here, the lesions extended to similar large sizes, and there were no major differences in lesion size between LJ43 and ZC108 at 2, 4 and 6 days after *C. camelliae* CCA inoculation. Based on the traditional lesion measurement method, both LJ43 and ZC108 were susceptible. This was different from previous reports on the anthracnose lesion sizes of LJ43 and ZC108 [10], perhaps because different *C. camelliae* strains were used in this study and a different wounding treatment was performed before inoculation.

However, when fungal biomass was measured, much more fungal growth occurred on LJ43 than on ZC108, as significantly more CCA DNA was detected in LJ43 by qPCR (Fig. 4c, d). This result indicates that the tea cultivar ZC108 was more resistant than LJ43 to *C. camelliae* CCA. Similar results were observed in previous studies [10, 37]. This also suggests that the growth of *C. camelliae* CCA was somehow restricted by ZC108, so it might have produced certain defence responses towards the fungal infection. Here, the qPCR results were different from the traditional lesion measurement method (Fig. 4b–d). This indicated that qRT-PCR analysis was more sensitive than the traditional lesion measurement method in the quantification of *C. camelliae* growth during its interaction with tea plant.

### DNA-based real-time PCR applied for analysis of tea LJ43 interaction with different *C. camelliae* isolates

**Fig. 5:**
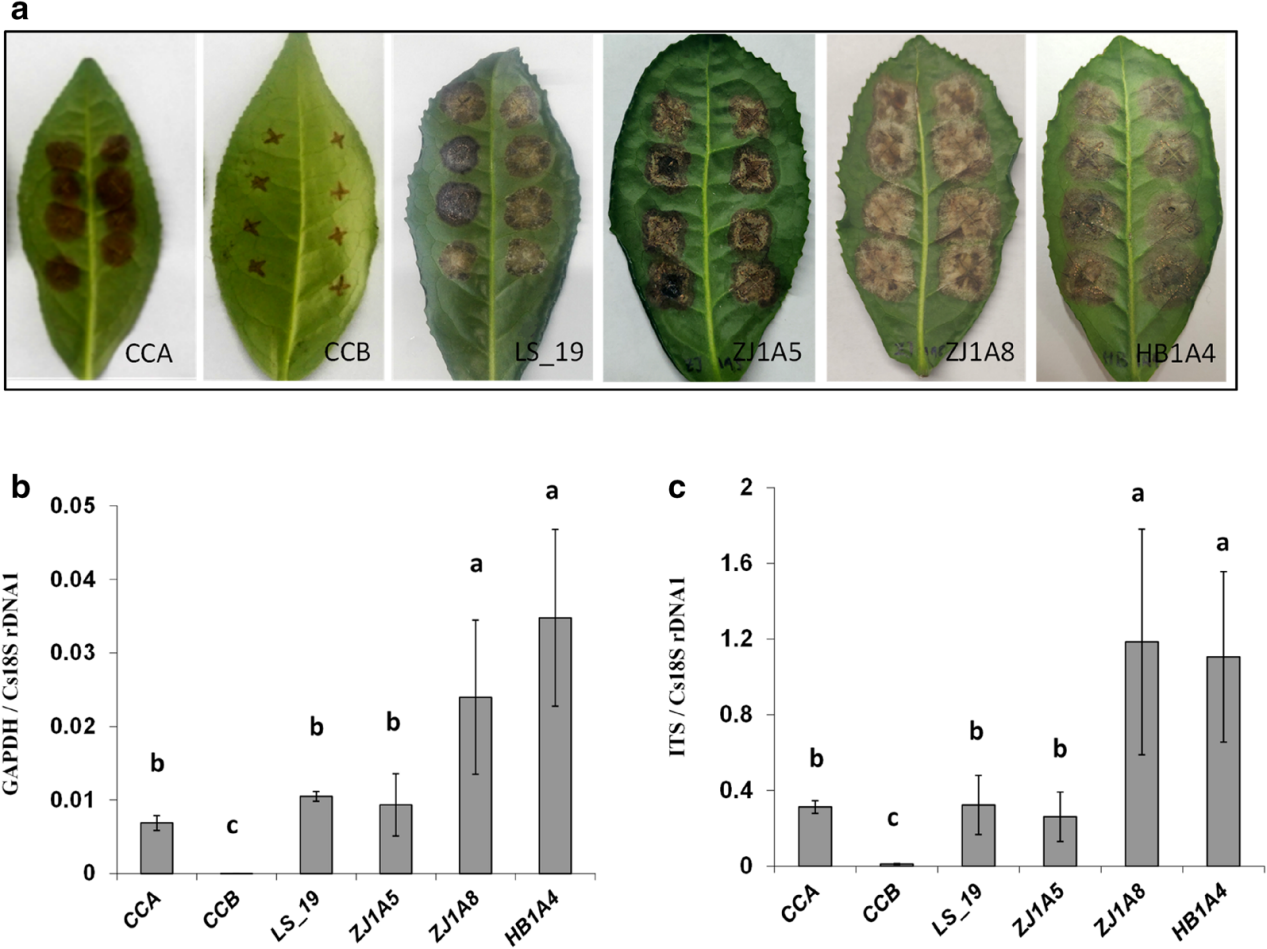
Quantification of different isolates of *C. camelliae* on tea plant cultivar LJ43. a Photos of anthracnose leaf symptoms developed 6 days post inoculation (6 DPI). CCA: *C. camelliae* CCA; CCB: *C. camelliae* CCB; LS_19: *C. camelliae* LS_19; ZJ1A5: *C. camelliae* ZJ1A5; ZJ1A8: *C. camelliae* ZJ1A8; HB1A4: *C. camelliae* HB1A4. **b**, **c** qRT-PCR-based biomass of six *C. camelliae* isolates grown on tea plants (6 DPI). **b** The ratio of the primer pairs GAPDH/Cs18SrDNA1 was used to determine fungal biomass. **c** The ratio of the primer pairs ITS/Cs18SrDNA1 was used to determine the fungal biomass. The letters a, b and c represent significant differences between the quantification of different isolates of *C. camelliae* according to LSD test (*P* < 0.05)

*Colletotrichum camelliae* is one of the dominant pathogens of tea in several provinces of China [21–24]. Previous reports have identified diverse *C. camelliae* isolates from various tea gardens in China [21–23]. Different fungal isolates can display distinct levels of aggressiveness towards their host plant. The *C. camelliae* isolates CCA, CCB, LS_19, ZJ1A5, ZJ1A8 and HB1A4 (Additional file 1: Table S1) were used to test differences in aggressiveness on tea cultivar LJ43 by qPCR assay. As shown in Fig. 5a, the leaf anthracnose symptoms produced by the different isolates of *C. camelliae* were very dissimilar. At 6 DPI, the lesion sizes of *C. camelliae* isolates CCA, LS_19 and ZJ1A5, on the infected tea plants were larger than those of CCB, while the largest lesion sizes were caused by ZJ1A8 and HB1A4 (Fig. 5a). Fungal growth biomass, as measured by qRT-PCR, revealed greater fungal growth in CCA-, LS_19- and ZJ1A5-infected plants than CCB, while the most fungal growth occurred for ZJ1A8 and HB1A4. The CCB-infected tea plants had very little fungal growth (Fig. 5b, c). These results indicated that ZJ1A8, HB1A4, CCA, LS_19 and ZJ1A5 were aggressive isolates, while CCB was not aggressive on the tea cultivar LJ43. This also was consistent with recent reports that *C. camelliae* LS_19 was a pathogenic isolate [23]. Here, *C. camelliae* isolates ZJ1A8 and HB1A4 were more aggressive than CCA, LS_19 and ZJ1A5. The qPCR method revealed the difference in aggressiveness among *C. camelliae* isolates on tea cultivar LJ43.

### DNA-based real-time PCR applied for the analysis of interactions between tea LJ43 and *Colletotrichum* spp*.*

**Fig. 6:**
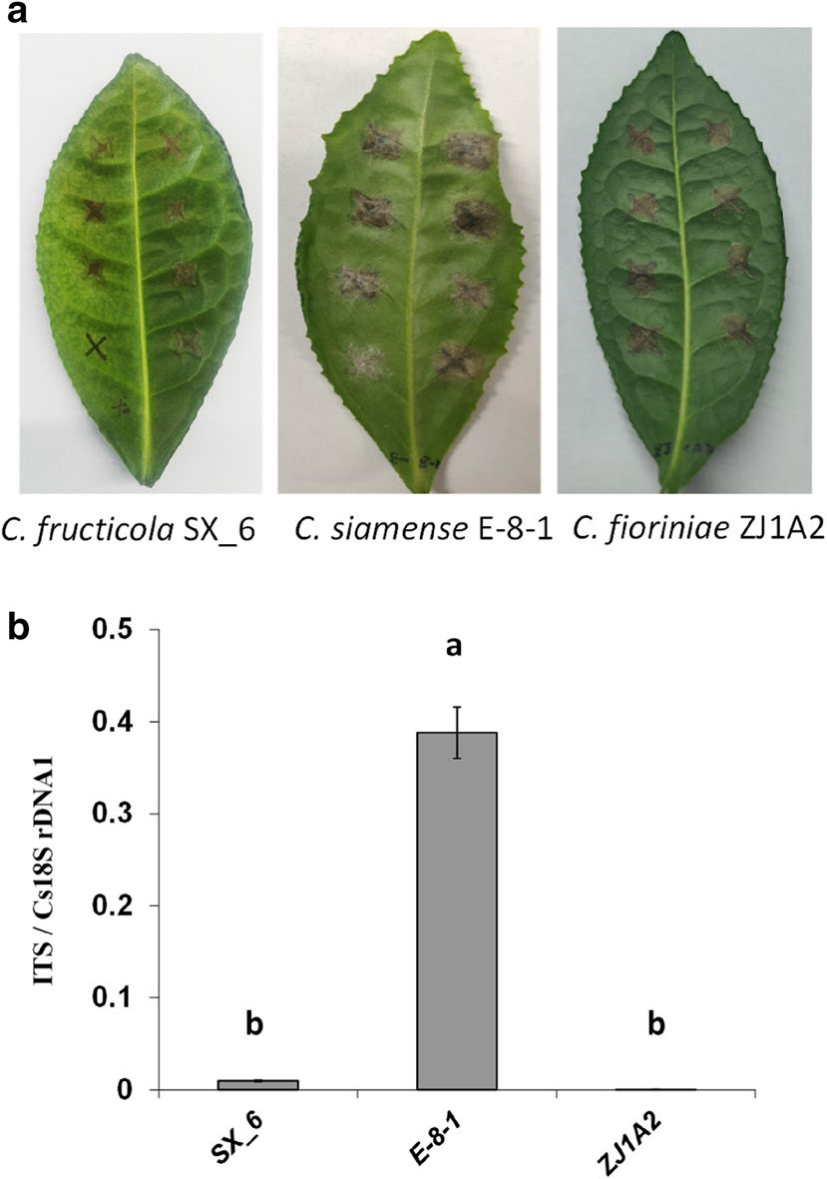
Quantification of different species of *Colletotrichum* on tea plant cultivar LJ43. **a** Photos of anthracnose leaf symptoms developed 6 days post inoculation (6 DPI). SX_6: *C. fructicola* SX_6; E-8-1: *C. siamense* E-8-1; ZJ1A2: *C. fioriniae* ZJ1A2. **b** qRT-PCR-based biomass of *Colletotrichum* spp. growth on tea plants (6 DPI). The ratio of the primer pairs ITS/Cs18SrDNA1 was used to determine the fungal biomass. The letters a and b represent significant differences between the quantification of different species of *Colletotrichum* according to LSD test (*P* < 0.05)

To compare the differences in the pathogenicity of *Colletotrichum* spp*.* on tea LJ43, *C. fructicola* SX_6, *C. siamense* E-8-1 and *C. fioriniae* ZJ1A2 also were examined. The ITS primer was used to test their growth differences. As shown in Fig. 6a, b, the leaf anthracnose symptoms and fungal growth differed among three species. *Colletotrichum siamense* E-8-1 infected tea plants producing larger lesion sizes and more fungal biomass than those inoculated with *C. fructicola* SX_6 and *C. fioriniae* ZJ1A2. This demonstrated that the qPCR method also can be used to compare and quantify the difference in aggressiveness of *Colletotrichum* spp*.* on tea plants.

## Discussion

Tea is a valuable crop because of its use in the beverage industry, and the use of tea as a medical crop has been demonstrated in many studies [1–3]. *Colletotrichum camelliae* is one of the dominant fungal pathogens of tea [22, 23]. The *C. camelliae*-tea interaction is a valuable pathosystem for determining how fungal pathogenicity is established and how tea plant defence responses are activated. These types of studies have created a critical need for new and accurate assays that measure small differences in *C. camelliae* growth in *planta*.

Visual quantification of lesion size can be used to distinguish differences in pathogen growth on very susceptible plants or when pathogens show large differences in aggressiveness. This method works less well for small differences in aggressiveness or when hosts display small differences in resistance [28, 30]. Recently, the qPCR methodology was shown to be a highly sensitive, reliable, simple and accurate way to quantify pathogen growth in other host–pathogen systems [28–30]. The qPCR procedures offer the added advantage of quantifying pathogen growth even at the early stages of the host-parasite interactions [28, 30].

In this study, a precise procedure was developed for qPCR quantification of *C. camelliae* growth on tea. This method included assays for both sides of the host-parasite interaction, one pathogen gene and one tea gene, which enables accurate normalization of the ratio between pathogen biomass and plant biomass (Figs. 2c, d; 3c, d; 4c, d; 5b, c; 6b). In these experiments, detached leaves were used, so plant tissue did not increase after inoculation. However, plant DNA might have been reduced as the host was damaged by certain pathogens at the infection sites. Thus, the amplification of the pathogen DNA sequence compared to that of the gene in the host plant gave a precise quantification of disease development and severity during the plant-pathogen interactions. In addition, the genomic DNA extraction and qPCR performance in this study were simple, rapid and the common reagents used in the experiments can be easily obtained in a laboratory.

A reliable PCR assay depends on having highly sensitive PCR primers that amplify the target DNA sequences. In the present study, the primers for two fungal targets, ITS and GAPDH sequences, were developed. The GAPDH primer was specific for amplifying *C. camelliae*, including all of its different isolates. The ITS primers designed here could detect both *C. camelliae* and its close relatives in the genus *Colletotrichum*. The ITS primer was better for quantification of *C. camelliae* growth during even the early stages of infection, when little pathogen growth had occurred. These primers did not detect other fungal pathogens, such as *P. camelliae-sinensis, Neopestalotiopsis* sp., or *M. oryzae*, which makes them useful for the quantification of *C. camelliae* growth on tea.

The Cs18SrDNA1 was used as target DNA sequence to monitor relative fungal growth in tea. The qPCR primers for amplification of tea Cs18SrDNA1 in this study was highly sensitive. The Cs18SrDNA1 has also been used as a reference gene to detect gene expression in tea [32]. Therefore, the primer for Cs18SrDNA1 can be used in studies of tea-*C. camelliae* interactions, not only to detect the expression of different genes, but also to help quantify relative pathogen growth.

It is estimated that more than 3000 tea accessions have been collected and conserved in the China National Germplasm Tea Repository [38]. Those genetic resources could provide diverse parental material for tea disease resistance breeding. It would be useful to test their responses to pathogens such as *C. camelliae*. Based on the qPCR method, *C. camelliae* growth differences were compared on the two national tea cultivars LJ43 and ZC108 in the current study. A previous infection assay revealed different responses to *C. camelliae* infection between ZC108 and its parent cultivar LJ43 by comparing the differences in lesion size [10]. While LJ43 was susceptible to *C. camelliae*-1 and *C. camelliae*-2, ZC108 was reported to be resistant to both [10]. Although in this study, the lesion measurement data indicated that both cultivars were highly susceptible to *C. camelliae* CCA, but the qPCR results indicated that ZC108 was more resistant than LJ43. In future, this qPCR assay could be used to screen the responses of other tea accessions towards *C. camelliae*.

Many studies have identified *Colletotrichum* spp. as the causal agents of several tea diseases [21–24, 39]. Previous research has shown that remarkable species diversity exists within the genus *Colletotrichum* that infect tea [21–23]. For example, different isolates of *C. camelliae* were collected from several provinces in China. In certain isolates, the conidiophores and setae were directly produced from the hyphae or on a cushion of roundish hyaline cells, while other isolates only produced aerial mycelium [22]. The genetic differentiation among *C. camelliae* isolates or *Colletotrichum* spp. with different morphology and aggressiveness phenotypes should be further clarified. The qPCR methodology developed in this study performed well in detecting differences in aggressiveness between *C. camelliae* isolates. Furthermore, this qPCR assay also could detect differences in aggressiveness among *C. camelliae* and its related species, such as *C. fructicola*, *C. siamense* and *C. fioriniae*.

## Conclusions

A procedure for the quantification of *C. camelliae* using qRT-PCR was studied as a new method for assessing the growth of this fungus on tea. This study indicated that the DNA-based qPCR assay was more sensitive and accurate than the traditional lesion measurement method. The qRT-PCR assay for assessing *C. camelliae* growth in tea plants was highly precise, sensitive and easily applicable, and this method could be used for identifying resistant tea plant cultivars or in screening for differences in pathogen aggressiveness.

## Materials and methods

### Plant materials and growth conditions

Tea *C. sinensis* cultivars Longjing 43 (LJ43) and Zhongcha 108 (ZC108) were used for all of the assays in this study. LJ43 was the parental cultivar of ZC108. Cuttings of LJ43 were irradiated by Co^60^γ-ray in 1986, and the mutant lines were further propagated for single-plant selection [37]. After a 24-year breeding procedure, one new line was selected and named ZC108. ZC108 was then registered as a new cultivar in China in 2010 [10, 37]. Two-year-old plants of LJ43 and ZC108 were grown in a disease-free climate chamber under 12 h light/12 h dark conditions at 25 ± 2 °C and 60% relative humidity before inoculation. For fungal inoculations, 50 mature leaves of 2-year-old LJ43 or ZC108 were randomly collected from more than 20 tea plants.

### Pathogen infection and fungal growth assay

*Colletotrichum camelliae* isolates CCA and CCB were both originally isolated from a diseased tea garden in Fancun, Hangzhou. Disease samples were collected from leaves showing visible anthracnose symptoms. The isolates were obtained by the single-spore isolation technique. The surface of leaves with anthracnose symptoms were first scratched with a small microbe-free blade and placed in sterilized water. After shaking for 20 min, the suspension was subjected to a tenfold dilution, and each dilution was distributed onto the surface of potato dextrose agar (PDA) culture medium (9.0 cm diameter Petri plates), followed by incubation in a climate chamber (22 ± 2 °C, 12 h light/12 h dark). Single germinated conidia were transferred to new PDA plates, and the incubation continued to generate the pure isolates.

The other tea anthracnose fungi including *C. camelliae* isolates (LS_19, ZJ1A5, ZJ1A8, and HB1A4), *C. fructicola* (SX_6), *C. siamense* (E-8-1) and *C. fioriniae* (ZJ1A2); the tea gray blight fungi such as *Pseudopestalotiopsis camelliae-sinensis* (HUN1A3) and *Neopestalotiopsis* sp*.* (YN1A5) were previously isolated from tea gardens and grown on PDA plates (Additional file 1: Table S1) [22, 23, 35]. *Magnaporthe oryzae,* which causes rice blast disease, was conserved on potato dextrose broth (PDB) medium and kept in the laboratory for the DNA assay.

All spores were cultivated, collected, washed, and frozen at ‒ 80 °C in 0.8% NaCl at a concentration of 10^8^ spores mL^−1^. For inoculation of tea plants, the spores were diluted in ddH_2_O. For droplet inoculations, 5 or 10 µL of 1 * 10^6^ spores mL^−1^ was applied to single detached tea leaves (2-year-old healthy tea plant cultivar LJ43 or ZC108) as previously described [40].

All of the inoculation experiments were designed as follows. For all treatments, leaves were wounded with a razor blade immediately before inoculation. Three replicates were carried out for each treatment. In each repetition, three to five mature leaves were used and each mature tea leaf usually received six to eight droplets of spores. For the control, ddH_2_O alone was used after wounding. All the leaves were completely randomly distributed during incubation. Each experiment was independently repeated at least three times.

During fungal inoculation, all detached leaves were consistently kept under sealed plastic hoods (Tianxing, Ningbo, China) at high humidity (> 80%). Inoculation was carried out on a bench at room temperature (around 25 °C); otherwise, the plants were placed into a specific climate chamber for fungal incubation under a strict light (12 h light/12 h dark) and temperature regime (25 ± 2 °C). After inoculation, the lesion size was visually measured and photographs were taken at different times (i.e., 1, 2, 3, 4, 5 and 6 days post infection). And then, leaves of similar size (which contained two or three lesions) were harvested and frozen at ‒ 80 °C for the DNA assays.

### DNA extraction

The DNA samples frozen in liquid nitrogen were homogenized using a TissueLyser (Qiagen, Hilden, Germany) for 2 × 30 s at 30 strokes/s A total of 400 µL of DNA extraction buffer (200 mM Tris–HCl, pH 7.5; 250 mM NaCl; 25 mM EDTA, and 0.5% SDS) was added to each of the homogenized samples, which were shaken again in the Tissue Lyser for 10 s at 30 strokes/s. The DNA extractions were the performed as previously described [40]. For each DNA sample, at least three technical replicates were performed.

### Quantitative real-time PCR

For qPCR analysis, approximately 30 ng of DNA was mixed with 0.4 mM gene-specific primers (GAPDH and ITS for the pathogen, while Cs18SrDNA1 was used for tea) (Table 1) and SYBR Green Supermix (Takara, Dalian, China) in a total volume of 25 µL. The reaction mixture contained 12.5 µL of SYBR Green, 1.5 µL of the forward and reverse primers, 9 µL of ddH_2_O and 2 µL of template DNA. qPCR was performed using the Applied Biosystems 7500 Sequence Detection System (ABI, Massachusetts, USA). The PCR program consisted of a preliminary step of 1 min at 95 °C followed by 40 cycles at 95 °C for 15 s and 60 °C for 34 s. A no template control for each primer pair was included in each run. The results were analysed using the Applied Biosystems 7500 software and Microsoft Office Excel based on the CT values observed. For each treatment, one representative set of results was presented as the mean 2^−∆∆CT^ value ± SEM. The relative amounts of pathogen DNA and tea DNA were determined by qPCR employing specific primers (Table 1).

### Statistical analysis

The lesion sizes and qPCR data were analysed with an analysis of variance (ANOVA) using the SPSS 18 software (IBM, New York, USA) and the least significant difference (LSD) test. All treatments were repeated independently three times. Reported values were presented as the mean ± standard error of three repeats, and a *P* value < 0.05 was considered statistically significant according to LSD test.

## Supplementary information

**Additional file 1: Table S1.** Geographical distribution of tea pathogens used in this research.

**Additional file 2: Figure S1.** Amplification results of target genes using DNA inputs from different organisms. M: DL2000 DNA Ladder (2000, 1000, 750, 500, 250, 100 bp); **a** Amplification results of GAPDH using DNA from *Colletotrichum camelliae* CCA (1), *C. camelliae* CCB (2), *C. camelliae* LS_19 (3), *C. camelliae* ZJ1A5 (4), *C. camelliae* ZJ1A8 (5), *C. camelliae* HB1A4 (6), *C. fructicola* SX_6 (7), *C. siamense* E-8–1 (8), *C. fioriniae* ZJ1A2 (9), *Pseudopestalotiopsis camelliae-sinensis* HUN1A3 (10), *Neopestalotiopsis sp.* YN1A5 (11), *Magnaporthe oryzae* (12) and tea cultivar LJ43 DNA control (13). **b** Amplification results of ITS using DNA from *Colletotrichum camelliae* CCA (1), *C. camelliae* CCB (2), *C. camelliae* LS_19 (3), *C. camelliae* ZJ1A5 (4), *C. camelliae* ZJ1A8 (5), *C. camelliae* HB1A4 (6), *C. fructicola* SX_6 (7), *C. siamense* E-8–1 (8), *C. fioriniae* ZJ1A2 (9), *Pseudopestalotiopsis camelliae-sinensis* HUN1A3 (10), *Neopestalotiopsis sp.* YN1A5 (11), *Magnaporthe oryzae* (12) and tea cultivar LJ43 DNA control (13).

## Data Availability

Not applicable.

## Abbreviations

qRT-PCR: Quantitative real-time polymerase chain reaction
EDTA: Ethylenediaminetetraacetic acid
SDS: Sodium dodecyl sulfate
