# Supplementary material for: Development of a DNA-based real-time PCR assay for the quantification of Colletotrichum camelliae growth in tea (Camellia sinensis)
Source: Plant Methods. 2020 Feb 17;16:17. doi: 10.1186/s13007-020-00564-x (PMC7027280; doi:10.1186/s13007-020-00564-x)
Supplement: Supplementary file 1 — Additional file 1: Table S1. Geographical distribution of tea pathogens used in this research. [file 13007_2020_564_MOESM1_ESM.docx]

| Tea pathogen species | Isolates | City | Province in China | Reference |
| --- | --- | --- | --- | --- |
| *Colletotrichum camelliae* | CCA | Hangzhou | Zhejiang | this study |
| *C.camelliae* | CCB | Hangzhou | Zhejiang | this study |
| *C.camelliae* | LS_19 | Lishui | Zhejiang | [23] |
| *C.camelliae* | ZJ1A5 | Hangzhou | Zhejiang | [22] |
| *C.camelliae* | ZJ1A8 | Hangzhou | Zhejiang | [22] |
| *C.camelliae* | HB1A4 | Enshi | Hubei | [22] |
| *C. fructicola* | SX_6 | Shaoxing | Zhejiang | [23] |
| *C. siamense* | E-8-1 | Hangzhou | Zhejiang | [22] |
| *C. fioriniae* | ZJ1A2 | Hangzhou | Zhejiang | [22] |
| *Pseudopestalotiopsis camelliae-sinensis* | HUN1A3 | Changsha | Hunan | [35] |
| *Neopestalotiopsis* sp. | YN1A5 | Lincang | Yunnan | [22] |

**Table S1. Geographical distribution of tea pathogens used in this research.**
