# Supplementary material for: Development of a DNA-based real-time PCR assay for the quantification of Colletotrichum camelliae growth in tea (Camellia sinensis)
Source: Plant Methods. 2020 Feb 17;16:17. doi: 10.1186/s13007-020-00564-x (PMC7027280; doi:10.1186/s13007-020-00564-x)
Supplement: Supplementary file 2 — Additional file 2: Figure S1. Amplification results of target genes using DNA inputs from different organisms. M: DL2000 DNA Ladder (2000, 1000, 750, 500, 250, 100 bp); a Amplification results of GAPDH using DNA from Colletotrichum camelliae CCA (1), C. camelliae CCB (2), C. camelliae LS_19 (3), C. camelliae ZJ1A5 (4), C. camelliae ZJ1A8 (5), C. camelliae HB1A4 (6), C. fructicola SX_6 (7), C. siamense E-8–1 (8), C. fioriniae ZJ1A2 (9), Pseudopestalotiopsis camelliae-sinensis HUN1A3 (10), Neopestalotiopsis sp. YN1A5 (11), Magnaporthe oryzae (12) and tea cultivar LJ43 DNA control (13). b Amplification results of ITS using DNA from Colletotrichum camelliae CCA (1), C. camelliae CCB (2), C. camelliae LS_19 (3), C. camelliae ZJ1A5 (4), C. camelliae ZJ1A8 (5), C. camelliae HB1A4 (6), C. fructicola SX_6 (7), C. siamense E-8–1 (8), C. fioriniae ZJ1A2 (9), Pseudopestalotiopsis camelliae-sinensis HUN1A3 (10), Neopestalotiopsis sp. YN1A5 (11), Magnaporthe oryzae (12) and tea cultivar LJ43 DNA control (13). [file 13007_2020_564_MOESM2_ESM.docx]

**a**

100bp

M 1 2 3 4 5 6 7 8 9 10 11 12 13

500bp

**b**

100bp

M 1 2 3 4 5 6 7 8 9 10 11 12 13

500bp

**Figure S1 Amplification results of target genes using DNA inputs from different organisms.** M: DL2000 DNA Ladder (2000, 1000, 750, 500, 250, 100 bp); **a** Amplification results of GAPDH using DNA from *Colletotrichum camelliae* CCA (1), *C. camelliae* CCB (2), *C. camelliae* LS_19 (3), *C. camelliae* ZJ1A5 (4), *C. camelliae* ZJ1A8 (5), *C. camelliae* HB1A4 (6), *C. fructicola* SX_6 (7), *C. siamense* E-8-1 (8), *C. fioriniae* ZJ1A2 (9), *Pseudopestalotiopsis camellia-sinensis* HUN1A3 (10), *Neopestalotiopsis sp.* YN1A5 (11), *Magnaporthe oryzae* (12) and tea cultivar LJ43 DNA control (13). **b** Amplification results of ITS using DNA from *Colletotrichum camelliae* CCA (1), *C. camelliae* CCB (2), *C. camelliae* LS_19 (3), *C. camelliae* ZJ1A5 (4), *C. camelliae* ZJ1A8 (5), *C. camelliae* HB1A4 (6), *C. fructicola* SX_6 (7), *C. siamense* E-8-1 (8), *C. fioriniae* ZJ1A2 (9), *Pseudopestalotiopsis camellia-sinensis* HUN1A3 (10), *Neopestalotiopsis sp.* YN1A5 (11), *Magnaporthe oryzae* (12) and tea cultivar LJ43 DNA control (13).
